# Higher Energy Demands and Lower Body Condition in a Hot Spring: Comparing Relict Leopard Frogs at Two Thermally Contrasting Sites

**DOI:** 10.1002/jez.70129

**Published:** 2026-09-01

**Authors:** Robert P. Pelletier, Michael D. Treat, Frank van Breukelen, Jef R. Jaeger

**Affiliations:** ^1^ School of Life Sciences, University of Nevada, Las Vegas Las Vegas Nevada USA

**Keywords:** body temperature, energy demands, hot spring, overwintering, temperature preference, thermoregulation

## Abstract

Following range‐wide decline, all remnant populations of the relict leopard frog (*Rana onca*) in the eastern Mojave Desert of the southwestern USA occupied geothermally influenced hot springs. In recent years, however, several large populations have been established at cold‐water sites. There has, however, been little research into the thermal ecology of the species. We monitored body temperature (T_b_) of adult frogs at a pair of hot and cold springs using implanted temperature data loggers, and measured body condition across seasons. We determined temperature preference using a thermal gradient, and measured mass‐specific oxygen consumption rates to estimate energy demands in the wild. We found that frogs at the two sites had similar temperature preferences, but the thermal profiles in the wild were seasonally 2.5°C to 5.7°C warmer at the hot site. In summer, frogs at the hot site mostly sought the lowest temperatures available, which were still warmer than measured thermal preferences. Frogs at the cold site maintained cooler temperatures at night, but warmed up during the early part of the day, likely moving in and out of cold water. At both sites, frogs in winter used two overwintering strategies, manifested mostly as differences in T_b_ variability. Frogs with low T_b_ variability thermally reflected cold water temperatures throughout most of winter and were interpreted as mostly inactive. Frogs with higher T_b_ and variability were interpreted as mostly remaining active throughout winter. We estimated that the energy demands at the hot site were 1.27‐fold higher in summer and 1.14‐fold higher in winter than at the cold site. Frogs at the hot site had significantly lower body condition than those at the cold site. We concluded that thermal conditions at the hot site were not favorable to *R. onca* and required higher energy investment in physiological maintenance, presumably at the expense of growth and reproduction.

## Summary

- Estimated energy demands for *Rana onca* at a hot spring were significantly higher than at a cold‐water site. Thermal conditions at the hot site were not favorable to *R. onca* and likely required high energy investment in physiological maintenance at the expense of growth and probably reproduction.

## 1 Introduction

Following substantial population decline, a species may be expected to persist in the most suitable habitat; however, if extirpated from once optimal locations, persistence may be possible in low‐quality habitat, at least for a while (Jansen and Yoshimura 1998; Leach et al. 2016; Britnel et al. 2022). The relict leopard frog (*Rana onca*) declined sometime during the 20th century, resulting in a substantial reduction of its historically narrow range in the eastern Mojave Desert of the southwestern USA (Bradford et al. 2004). By 2001, the few remnant sites occupied by the species were all geothermally influenced hot springs with source water temperatures > 30°C (Bradford et al. 2004). The persistence at hot springs may have resulted from the failure of invasive predators like the red swamp crayfish (*Procambarus clarkii*) and American bullfrog (*Rana catesbeiana*) to colonize these sites (Bradford et al. 2004). Another possibility was that the hot springs provided some level of protection from the invasive amphibian chytrid fungus (*Batrachochytrium dendrobatidis*; Forrest and Schlaepfer 2011; Jaeger et al. 2017); the upper thermal limit for this pathogenic fungus is generally close to or below the source temperatures of the hot springs (Piotrowski et al. 2004; Hettyey et al. 2019). Warm conditions at the hot springs, however, may instead have simply benefited *R. onca* by providing thermal refuge from cold winter temperatures in the eastern Mojave Desert.

Higher temperatures increase the energy demands of ectotherms and can be detrimental in winter when many animals are reliant on energy reserves (Sinclair et al. 2013; Tattersall and Ultsch 2008). In cold‐temperate regions, ectotherms can mitigate energetic demands by overwintering at cold temperatures, remaining relatively inactive (Donohoe et al. 1998). *Rana onca* has often been observed to be active during winter, including occasional breeding (Bradford et al. 2005; Rivera et al. 2023). Winter activity suggests that *R. onca* may not follow the overwintering strategy common to anuran species in cold‐temperate regions, instead potentially maintaining low temperatures only intermittently, if at all.

Summer also presents an energetic challenge for *R. onca* at hot springs. In the eastern Mojave Desert, summer ambient temperatures can be extreme; the long‐term mean daily high temperature in summer at a site near springs occupied by *R. onca* was 42.9°C (June–August, 1991–2020; NOAA 2025). While the upper thermal tolerance for adult *R. onca* has not been determined, frogs at hot springs have mostly been observed in colder‐water microhabitats, as well as lower reaches of streams where temperatures were cooler, suggesting potential avoidance or mitigation of hot temperatures.

As a species of conservation concern, *R. onca* is under multiagency management (U.S. Fish and Wildlife Service 2016, 81 Fed. Reg. 69434). One of the main management actions has been the establishment of new populations through translocations, mostly at cold‐water sites, using headstarted animals sourced from the remnant populations at hot springs. The largest populations of *R. onca* are now at cold‐water sites, indicating favorable conditions. Despite success in cold water, we do not know whether hot water temperatures at the remnant sites are generally beneficial or detrimental to this frog, particularly when ambient temperatures are at seasonal highs and lows.

To better understand the thermal ecology of *R. onca*, we used temperature data loggers to monitor the body temperature (T_b_) of adult frogs in the wild for almost 2 years at a pair of hot and cold spring systems. We determined temperature preferences of wild‐caught adult frogs using a thermal gradient. We measured mass‐specific oxygen consumption rate (V̇O_2_) in the laboratory to estimate energy demands in the wild. At both sites, we monitored adult body condition across seasons. Here, we provide a portrait of the energetic demands and thermal opportunities for *R. onca* frogs at both hot and cold spring sites, concluding that cold pools and seeps at the hot site are critical for the persistence of the population.

## 2 Materials and Methods

### 2.1 Study Sites

We selected Boy Scout Canyon within Black Canyon, Clark County, Nevada as the hot spring study site (hereafter hot site). The narrow canyon, at an elevation of ~200–313 m, contained a relatively small, remnant population of *R. onca*. A hot stream flows through the canyon into the Colorado River, fed by springs with source water temperatures as high as 55°C (Pohlmann et al. 1998; Bradford et al. 2004; Beard et al. 2013). The hot site also contained several cold‐side pools and seeps, as well as an inaccessible cold‐water side stream. For the cold spring study site (hereafter cold site), we selected Union Pass Spring in the Black Mountains, Mohave County, Arizona, at an elevation of ~1128 m at the source. This site contained a relatively large population of *R. onca* established through translocations from 2011 to 2015. These frogs primarily occupied a perennial stretch of cold stream.

### 2.2 Body Temperatures

We implanted temperature loggers (iButton Model DS1925L; Dallas Semiconductor, Inc.) into the abdominal cavity of larger frogs (≥ 54 mm snout‐vent length, SVL) to continuously monitor T_b_ (every 5 min). We retrieved the loggers after monitoring individual frogs for various periods of 1.0 to 14.2 months (mean 5.7 months), recovering data from 12 of 19 frogs implanted at the hot site and 17 of 35 frogs implanted at the cold site. Collectively, we monitored frogs at the hot site from December 21, 2022 to October 14, 2024, and at the cold site from July 20, 2022 to July 10, 2024.

We followed the procedure for implanting temperature loggers in abdomens of frogs described by Rausch et al. (2008). The loggers were dipped in parafilm and weighed ~3.1 g, averaging 13.1% of the body mass of individual implanted frogs (mean body mass = 26.3 g). We allowed frogs an average of 5 days for recovery and confirmed feeding on small crickets prior to release of the frogs at points of capture. For T_b_ analyses we joined corresponding seasonal data from the different years to bolster sample sizes. We used a linear mixed‐effect model in R (R Core Team 2024; package nlme, Pinheiro et al. 2023) with a random effect for frogs to account for differences in sample sizes, and potential correlations among observations from repeated measurements or shared environmental conditions. We assessed the resulting data with a likelihood‐ratio test (R package car, Fox and Weisberg 2019) to compare the mean daily T_b_ of frogs across seasons and between the two sites. The test statistic was approximated by a *χ* ^2^ distribution. We used a post hoc Tukey's honestly significant difference test (HSD; R package emmeans, Lenth 2025) for pairwise comparisons among variables.

### 2.3 Environmental Reference Temperatures

For reference, we monitored environmental temperatures in areas occupied by *R. onca* using temperature data loggers sealed in paraffin and plastic. At the hot site, we recorded water temperatures in two cold pools, a warm pool, and the hot‐water stream (Supporting Information S1: Table S1). At the cold site, we recorded water temperatures in four pools within the cold‐water stream (there were no side pools). We recorded ambient air temperatures at several shaded locations adjacent to the streams at both sites.

### 2.4 Seasonal Patterns

We defined seasons based on temperature patterns as winter (December–February), spring (March–May), summer (June–August), and fall (September–November). We observed two apparent winter patterns in T_b_ at both sites, mostly corresponding to high and low variability among individual frogs. To parse these two patterns, we averaged the difference in daily temperature (maximum to minimum) of each frog, and then used k‐mean cluster analysis (R package cluster, Maechler et al. 2023) to identify two groups at each site. We then compared T_b_ and variability between the two groups and sites with a likelihood‐ratio test using frogs as a random effect, after adjusting the mean daily T_b_ using a linear mixed‐effect model, as above.

Our study design was not intended for statistical analysis of within‐season patterns of T_b_ or the potential in‐season influences of air and water temperatures. Even so, specific *post‐hoc* patterns in T_b_ within seasons were readily discernable amongst frogs, especially when viewed over diel (sunrise to sunrise), daytime (sunrise to sunset), and nighttime (sunset to sunrise) periods. For each period, we determined whether T_b_ of each frog was predominately within 2°C of cold‐water temperature or more aligned with air temperature, as determined from environmental loggers near the frog's capture location. We then calculated the percentage of periods during which each frog exhibited these different T_b_ patterns. We grouped frogs from each site according to their predominant pattern, and computed means and standard deviations for each group.

### 2.5 Temperature Preference

We assessed potential winter and summer differences in temperature preferences of wild‐caught frogs from the hot and cold sites using a linear thermal gradient. We followed a protocol similar to that described by Sanabria et al. (2020). In a thermal chamber, 1.8 m long and 10 cm wide (enough for frogs to freely turn around), we maintained a gradient from ~10 to > 40°C along an aluminum floor covered with a wet cotton cloth. We monitored temperature every 5 min using 24 evenly spaced temperature loggers attached to the floor, and interpolated temperatures between the loggers. We ran trials in a lighted room during daylight hours within 5 days of animal capture, measuring frog mass directly before each trial. We monitored each frog for 3 h by video recording, discarding the first hour to allow frogs to adjust to the gradient. We determined frog location (thus temperature) at 5‐min intervals based on the posterior of the frog's urostyle. We assessed 16 frogs captured from each site during winter (2023–2024) and again in summer (2024; 64 frogs total). We attempted to use only males to avoid gravid females (a potential confounding factor), but to reach sample size at the hot site during summer, we used 4 females who did not appear to be gravid.

We used a two‐way Analysis of Covariance (ANCOVA) to compare the temperature preference of frogs, with site and season as variables and mass as a covariable (Witters and Sievert 2001). We assessed for preference or avoidance of specific temperatures using the approach described by Verbitsky et al. (2018), implemented in R (packages dplyr and tidyr). We placed all frog observations from the temperature selection trials (5‐min intervals) into 1°C bins from 10°C to 40°C (with the highest bin defined as ≥ 40°C). We then categorized bins by observation frequency, with the central 70% identified as preferred, the surrounding 20% considered expected (occasional), and the outlying 10% as avoided (Verbitsky et al. 2018).

### 2.6 Modeling of Energy Demands

We measured mass‐specific V̇O_2_ of adult male *R. onca* in response to environmentally relevant temperatures. We captured 16 frogs of similar size from the cold site in summer and again in winter (32 total), maintaining the summer group at 22°C and the winter group at 10°C prior to V̇O_2_ measurements (trials). We ran three experimental 180 mL chambers, with two containing frogs and the third an empty control. These chambers permitted only limited movement of the frogs. The chambers were connected to a Field Metabolic System FMS (Sable Systems, Las Vegas, Nevada, USA) that monitored V̇O_2_ at an air flow rate of 200–250 mL•min^−1^ (through the chambers). The chambers were maintained at temperature within a closed box with an outer jacket through which water circulated from a temperature‐controlled bath. To avoid desiccation of frogs, we placed a moist cotton cloth in each chamber and humidified inflowing air with a sodium chloride saturated solution. We dehumidified air after it left the chambers using Drierite prior to entering the FMS. We ran trials at 5, 15, 25, and 35°C, over 2‐h periods, with each frog measured at each trial temperature within 8 days of capture following a randomized design. We fasted frogs during this period, and measured mass prior to initial trials. We discarded the first hour of data to allow stabilization. We compared rates across the various temperatures for the frogs collected in winter and summer using a two‐way Analysis of Variance (ANOVA; Gray et al. 2016), followed by a post hoc Tukey's HSD.

We modeled seasonal energy demands using the mean V̇O_2_ of frogs at each trial temperature to calculate Q_10_ values (temperature coefficients) between the 10°C trial increments. We used these Q_10_ values to estimate V̇O_2_ values for mean T_b_ of frogs monitored in the field for each season and site. For these estimations, we matched laboratory V̇O_2_ data obtained from frogs collected in summer to field T_b_ data from summer, and followed the same approach for winter. For spring and fall, we averaged summer and winter laboratory V̇O_2_ data to use in estimates.

We compared estimates of energy demand at the hot and cold sites to determine differences in the energy costs associated with seasonal temperatures. We used linear mixed‐effect models following the framework described above to correct for unbalanced sample sizes. Using the adjusted data, we compared mean daily V̇O_2_ of frogs between hot and cold sites among seasons with a likelihood‐ratio test approximated by a chi‐square distribution. We followed the test with post‐hoc Tukey's HSD. To quantify overall seasonal differences in estimated energy demands between sites, we derived Q_10_ values from the modeled average V̇O_2_ values of frogs at each site per season.

### 2.7 Body Conditions

We measured body conditions of adult frogs approximately every 2 months at the hot site from December 13, 2022 to September 7, 2024 and at the cold site from November 28, 2022 to September 2, 2024. We recorded sex, and measured both body mass and SVL. To keep track of repeated captures, we marked adult frogs (≥ 45 mm SVL) at first capture by inserting a Passive Integrated Transponder tag (Biomark, Boise, Idaho) subcutaneously following established protocols (see Bradford et al. 2004). We separately assessed overall size (SVL) and body conditions of individual frogs by sex from each sampling event. For body condition, we used the Scaled Mass Index (SMI) that accounts for allometric relationships between mass and SVL (Peig and Green 2009; Ruthsatz et al. 2019). For statistical comparison, we used a linear mixed‐effect model following the framework described above, and analyzed the adjusted data with a likelihood‐ratio test. We compared SMI of adult frogs using the first measurement of each frog as a covariant and fixed factors of sex, site, and season (Liess et al. 2013), followed by a post hoc Tukey's HSD. We calculated differences in SVL in adult frogs using the same statistical approach.

## 3 Results

### 3.1 Body Temperatures Vary Between Sites and Seasons

We found that frog T_b_ at the hot site was higher across seasons than at the cold site (likelihood‐ratio test: site *χ* ^2^_1,1_ = 19.61, *p* < 0.01; season *χ* ^2^_3,1_ = 1037.08, *p* < 0.01; Figure 1). There was no interaction between site and season (likelihood‐ratio test: *χ* ^2^_3,1_ = 5.65, *p* = 0.13). During summer, mean T_b_ was 5.69°C higher at the hot site than at the cold site (Tukey's HSD: *p* < 0.01). During winter, the mean T_b_ was 2.48°C higher at the hot site (Tukey's HSD: *p* < 0.01). Differences between the sites were emphasized by the observations that T_b_ at the cold site in summer did not differ from that at the hot site in fall (Tukey's HSD: *p* = 0.40), and T_b_ at the hot site in winter did not differ from that at the cold site in spring (Tukey's HSD: *p* = 0.99). All other seasonal comparisons were significantly different (Tukey's HSD: *p* < 0.01).

**Figure 1:**
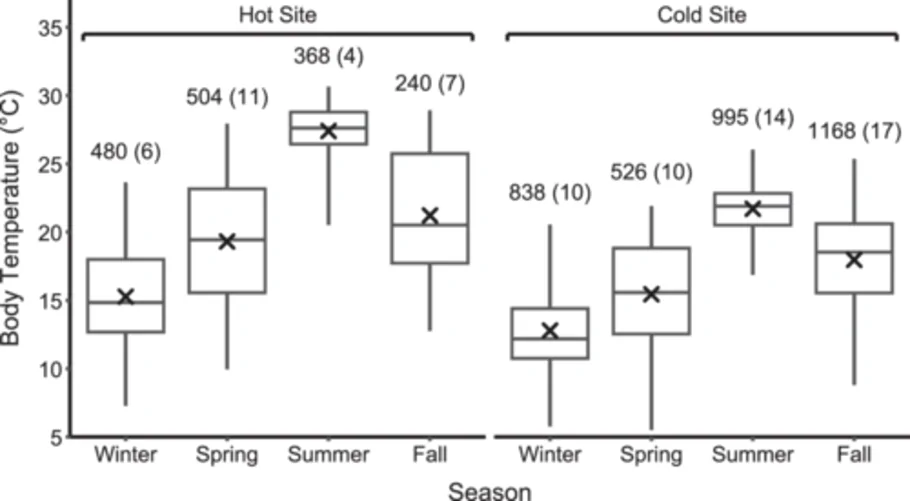
Mean daily body temperatures of adult *Rana onca* by season and site (hot and cold springs). The boxplots indicate the 25th and 75th percentiles, with medians depicted by bold horizonal lines and extent represented by vertical lines. The mean of body temperatures for each group is indicated by an X. Samples sizes above each plot represent the number of daily averages, with the number of frogs contributing data shown in parentheses.

### 3.2 Summer Diel Body Temperature Patterns

In summer at the hot site, mean T_b_ of frogs was 27.41°C ± 1.89°C and showed little variability between daytime and nighttime periods. The T_b_ was substantially below the temperature of the hot stream, as well as below the mean air temperature (Supporting Information S1: Table S1). We observed that T_b_ was mostly at, or between, the temperature of the two cold pools (Figure 2).

**Figure 2:**
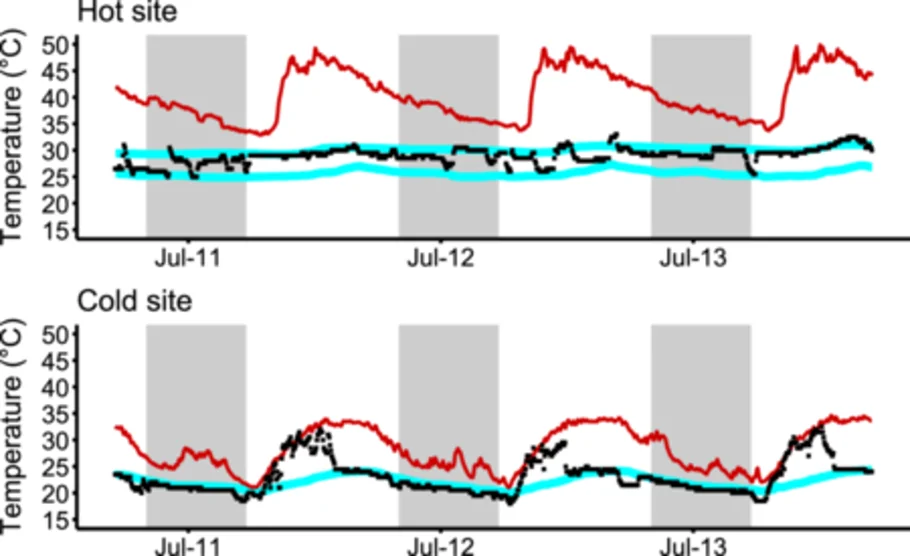
Three‐day snapshot of *Rana onca* body temperature in summer (July 11–13) from a representative frog (black points) at a hot spring and a representative frog at a cold spring, along with temperatures from representative cold‐water pools (thick light blue lines) and ambient air temperatures (thin dark red lines). The hot site includes temperatures from two cold pools, demonstrating the range of cold‐water temperatures at that site. Shading indicates times between sunset and sunrise. Data were recorded at 5‐min intervals.

At the cold site in summer, the diel T_b_ generally showed more variability between day and night than at the hot site (Figure 2). The mean nighttime T_b_ of these frogs was 20.66°C ± 1.48°C and reflected the temperature of the cold‐water stream at the site, but then rose with air temperature at sunrise until the afternoon (around 1300 to 1600 PDT) when T_b_ reached a mean of 28.28°C ± 3.81°C. Frog T_b_ then dropped abruptly back to roughly that of the stream temperature (Figure 2), while air temperature generally continued to rise into the late afternoon. This diel pattern was followed by 69% (9/13) of the frogs at the cold site over the majority (77%) of their diel observations in summer. The other 31% (4/13) of the frogs at the cold site had T_b_ that appeared to track the temperatures of the cold‐water stream most of the time (74% of their diel observations). Each frog, however, would switch to the opposing pattern for periods ranging from 1 to 15 days.

### 3.3 Winter Diel Body Temperature Patterns

We differentiated frogs into two groups in winter based primarily on clustering of T_b_ variability. We identified four frogs at the hot site and five at the cold site with high‐variability (hereafter H‐V frogs), and two frogs at the hot site and five at the cold site with low‐variability (hereafter L‐V frogs). We found differences in winter T_b_ between the two groups (likelihood‐ratio test: *χ* ^2^_1,1_ = 14.19, *p* < 0.01) and between sites (*χ* ^2^_1,1_ = 5.69, *p* = 0.02). The mean daily difference between maximum to minimum T_b_ of H‐V frogs was 7.62°C ± 4.11°C at the hot site and 4.83°C ± 2.77°C at the cold site. In comparison, these values for L‐V frogs were 2.93°C ± 2.86°C and 2.88°C ± 1.37°C, respectively. H‐V frogs in winter also had higher mean T_b_ than the L‐V frogs at both the hot site (H‐V frogs = 16.62°C ± 2.91°C; L‐V frogs = 12.56°C ± 3.05°C) and cold site (H‐V frogs = 14.52°C ± 3.00°C; L‐V frogs = 11.11°C ± 1.59°C).

In winter at the hot site, daytime T_b_ of most H‐V frogs (77% of daytime observations) averaged 14.93°C ± 2.19°C, and appeared to roughly track the temperature of one or the other of the cold pools at the site (Figure 3). On the other winter days, thermal profiles suggest that H‐V frogs occupied either sun‐exposed areas or more likely thermally influenced areas associated with the hot stream. At those times, the mean daytime T_b_ was 20.70°C ± 2.67°C, well above that of the cold pools and the recorded daytime air temperature in winter (13.23°C ± 3.11°C). At night, T_b_ of most H‐V frogs (73% of nighttime observations) averaged 18.32°C ± 3.77°C, above the cold‐water temperatures and the recorded nighttime winter air temperature (12.36°C ± 2.73°C; Figure 3). On the other nights, H‐V frogs at the hot site appeared to track the average temperature of the two cold pools for ≤ 14 days, maintaining a mean T_b_ of 14.06°C ± 1.76°C. The two L‐V frogs at the hot site spent all but eight and 10 days of winter, respectively, at a mean temperature of 11.62°C ± 1.65°C, within the temperature range of the coldest pool at the site. During the interludes, T_b_ reflected the warm and active pattern seen in H‐V frogs.

**Figure 3:**
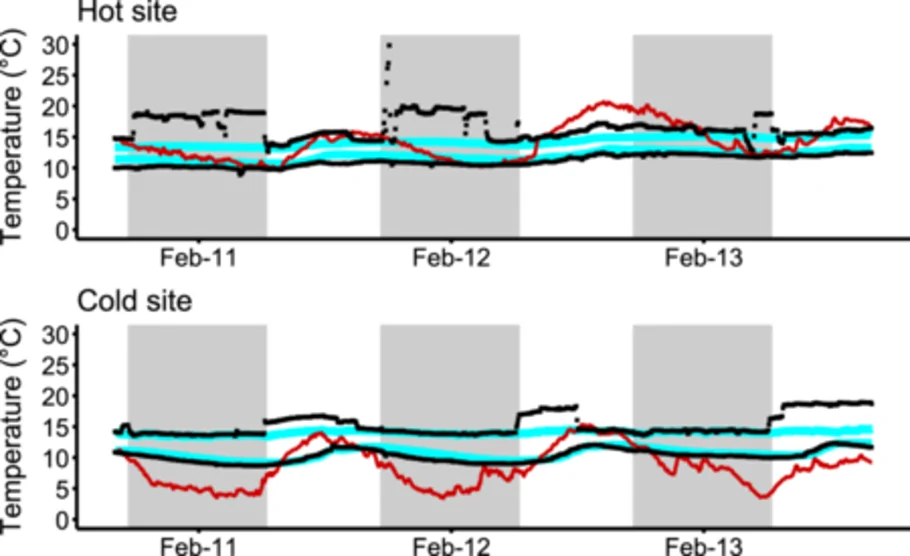
Three‐day snapshot of *Rana onca* body temperature in winter (February 11–13) from representative frogs (black points) at a hot and a cold spring, along with temperatures from representative cold‐water pools (thick light blue lines) and ambient air temperatures (thin dark red lines). Each graph shows body temperature from two frogs representing different overwinter strategies (high‐variability and low‐variability frogs), relatively active (upper black points) and relatively inactive (lower black points). Water temperatures are shown from two cold pools, demonstrating the range of cold‐water temperatures. Shading indicates times between sunset and sunrise. Data were recorded at 5‐min intervals.

At the cold site during winter, H‐V frogs mostly had lower nighttime than daytime T_b_ (74% of observations; Figure 3). The mean nighttime T_b_ of these frogs was 13.57°C ± 2.50°C, and their daytime mean T_b_ was 15.19°C ± 2.30°C. The nighttime thermal profiles appeared to track the cold‐water pools, whereas the daytime T_b_ was higher than water temperature and exceeded the daytime winter air temperature of 7.48°C ± 3.58°C. We suspect that daytime T_b_ at these times reflected activities outside of the stream influenced by solar radiation. On occasions, some H‐V frogs at the cold site in winter reached T_b_ that averaged 19.20°C ± 1.96°C for up to 10 days (10% of diel observations). Possibly these frogs accessed a ground water source or deep‐soil thermally stable microhabitat during these periods. All H‐V frogs spent short periods of up to 12 days with diel T_b_ of 13.18°C ± 1.08°C, closer to that of the cold‐water pools (16% of diel observations). The mean T_b_ of L‐V frogs in winter at the cold site was 11.09°C ± 1.68°C, which essentially tracked the coldest water at the site (Figure 3). These L‐V frogs maintained such temperatures through almost the entirety of the winter (99% of observations).

### 3.4 Fall and Spring Diel Body Temperature Patterns

Most of our capture events for implantation and retrieval of temperature data loggers occurred during spring and fall months, resulting in mostly incomplete or limited T_b_ data sets for these seasons. Even so, the temperature patterns of frogs in the fall season at both sites generally appeared to be extensions of summer patterns until frogs transitioned to winter patterns. This was relatively abrupt for frogs that switched to low‐variability patterns in winter (L‐V frogs), which occurred mostly in mid‐September through October at the cold site and in October and mid‐November at the hot site. Other frogs showed a steady decline in daily maximum T_b_, as well as a damping of temperature variability seen in summer, until reaching the winter pattern seen for H‐V frogs. The spring pattern was essentially reversed at both sites, with L‐V frogs starting to transition from the winter pattern sometime during March through mid‐April.

### 3.5 Temperature Preference

We found no difference in temperature preference between frogs from the hot and cold sites collected during either summer or winter (two‐way ANCOVA: site *F*_1,59_ = 0.23, *p* = 0.64; season *F*_1,59_ = 2.12, *p* = 0.15). During summer, frogs from the hot and cold sites selected very similar mean temperatures of 21.62°C ± 2.52°C and 21.00°C ± 6.07°C, respectively (Figure 4). In winter, frogs from the hot site selected a mean temperature of 20.48°C ± 5.67°C, while frogs from the cold site selected a mean temperature of 18.76°C ± 2.76°C (Figure 4). Frog mass did not affect temperature preference (two‐way ANCOVA: *F*_1,59_ = 0.16, *p* = 0.69). Given little difference in temperature preference between sites and seasons, we pooled the data for temperature binning prior to the determination of preferred and avoided temperatures. Frogs preferred temperatures of 13°C to 24°C, occasionally used 11°C to 12°C and 25°C to 27°C, and avoided temperatures of 10°C and ≥ 28°C (Figure 5).

**Figure 4:**
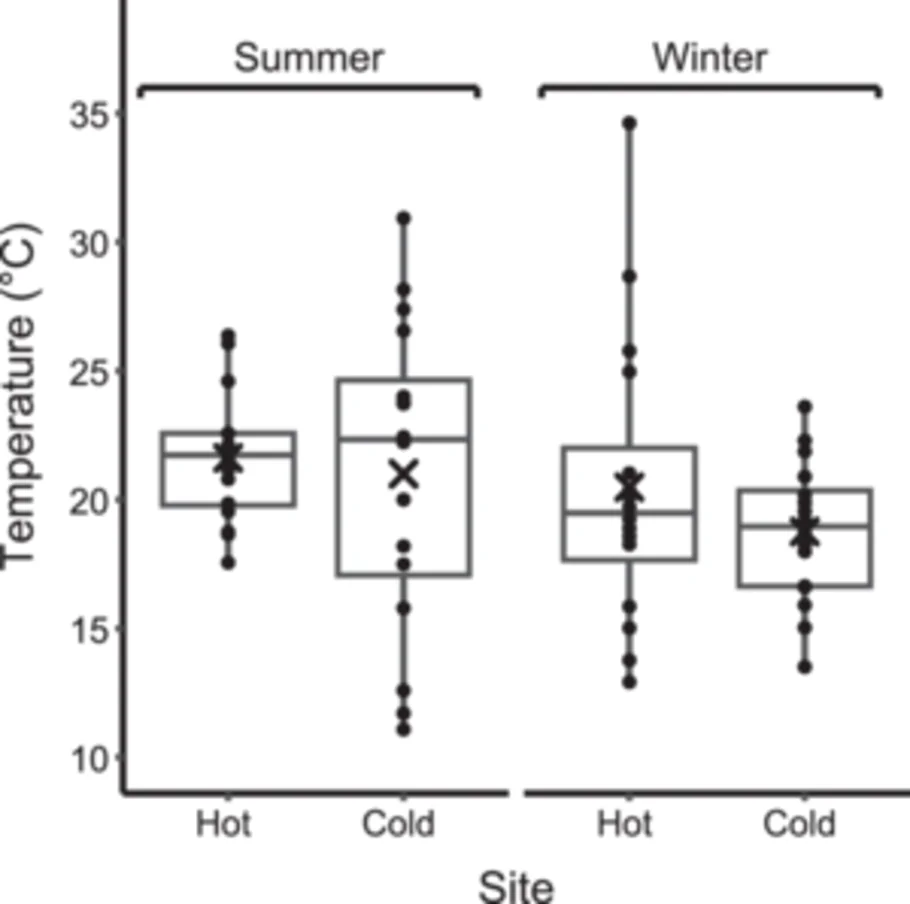
Boxplot of mean temperatures selected on a thermal gradient by adult *Rana onca* collected from a hot and a cold site during summer and winter. Data points represent the mean temperature selection of a frog, calculated from temperature recorded at 5‐min intervals over a 3‐h trial, with the first hour discarded. The boxplots indicate the 25th and 75th percentiles of each group, with medians indicated by bold horizonal lines, means indicated by Xs, and extent of data represented by vertical lines.

**Figure 5:**
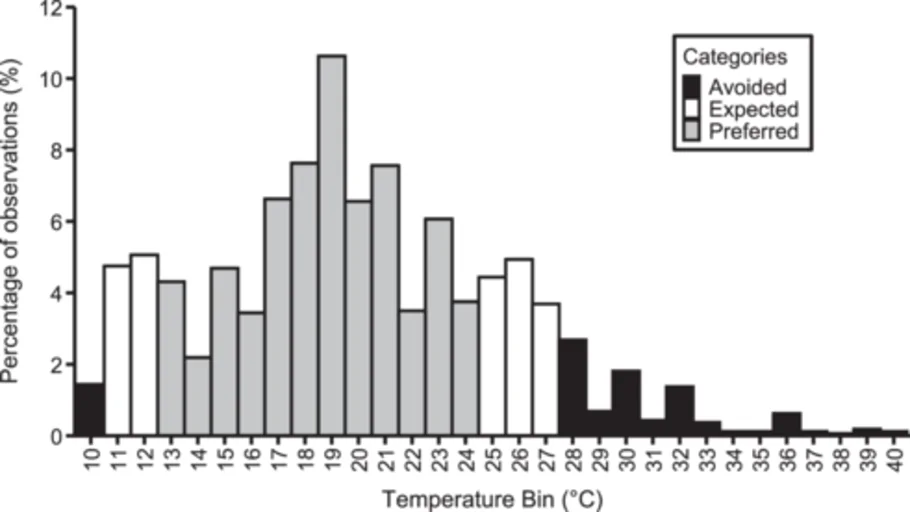
Temperature selection by 64 adult *Rana onca* on a thermal gradient grouped into 1°C bins (e.g., 30 = 30.0–30.9) by percentage of time. Bins are categorized as preferred (grey), expected/occasional (white), and avoided (black). The highest bin was defined as ≥ 40°C. Temperatures were recorded at 5‐min intervals over 3‐h trials, with the first hour discarded. Frogs were collected from a pair of hot and cold sites during summer and winter.

### 3.6 Energy Demands

The V̇O_2_ of adult frogs measured in the laboratory differed across the trial temperatures (ANOVA: *F*_3,1_ = 40.45, *p* < 0.01), as well as between the winter and summer samples (ANOVA: *F*_1,1_ = 5.08, *p* = 0.03). V̇O_2_ increased with temperature in both seasonal samples, with the winter values higher than the summer values for a given temperature. The associated Q_10_ decreased with increased temperature in summer, but showed no trend in winter (Figure 6). The difference in Q_10_ between sites was greatest in summer.

**Figure 6:**
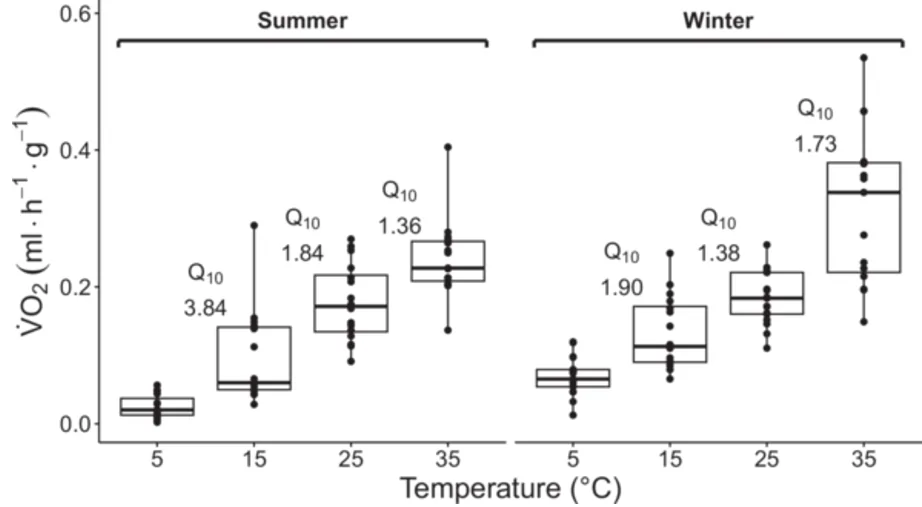
Mass‐specific oxygen consumption rates (V̇O_2_) measured from adult *Rana onca* captured during summer and winter by trial temperature. Q_10_ calculated for each 10°C interval are shown. Points represent the V̇O_2_ of individual frogs. The boxplots indicate the 25th and 75th percentiles, with medians depicted by bold horizonal lines and extent of data represented by vertical lines.

The estimated energy demands of frogs at the hot site were higher than those at the cold site (likelihood‐ratio test: *χ* ^2^_1,1_ = 14.72, *p* < 0.01) across seasons (*χ* ^2^_3,1_ = 481.70, *p* < 0.01; Supporting Information S1: Table S2). At the hot site, the estimated energy demands of frogs during summer were higher than in all other seasons (Tukey HSD: all *p* < 0.01), while energy demands at the cold site during winter were lower than in all other seasons (Tukey HSD: all *p* < 0.01). Energy demands during summer at the cold site were not different from those during fall at the hot site (Tukey HSD: *p* = 0.80), and energy demands during winter at the hot site were not different from those of spring at the cold site (Tukey HSD: *p* = 0.98).

### 3.7 Body Condition

Adult frogs at the hot site had lower SMIs than those at the cold site (likelihood ratio test: *χ* ^2^_1,1_ = 205.48, *p* < 0.01; Figure 7). There was also a difference in SMI between the sexes (likelihood ratio test: *χ* ^2^_1,1_ = 47.59, *p* < 0.01), but this difference was manifested only at the cold site where females had significantly higher SMIs than males (Tukey's HSD: *p* < 0.01; Supporting Information S1: Table S3). The pattern in body condition was reflected in body size. Adult frogs at the hot site had significantly smaller SVLs than those at the cold site (likelihood ratio test: *χ* ^2^_1,1_ = 4.45, *p* = 0.03), and SVLs of the sexes differed (likelihood ratio test: *χ* ^2^_1,1_ = 15.92, *p* < 0.01). The difference in SVL was only at the cold site where females were significantly larger than males (Tukey's HSD: *p* < 0.01). Body condition and body size also differed among seasons (likelihood ratio tests: SMI, *χ* ^2^_3,1_ = 30.16, *p* < 0.01; SVL, *χ* ^2^_3,1_ = 22.66, *p* < 0.01).

**Figure 7:**
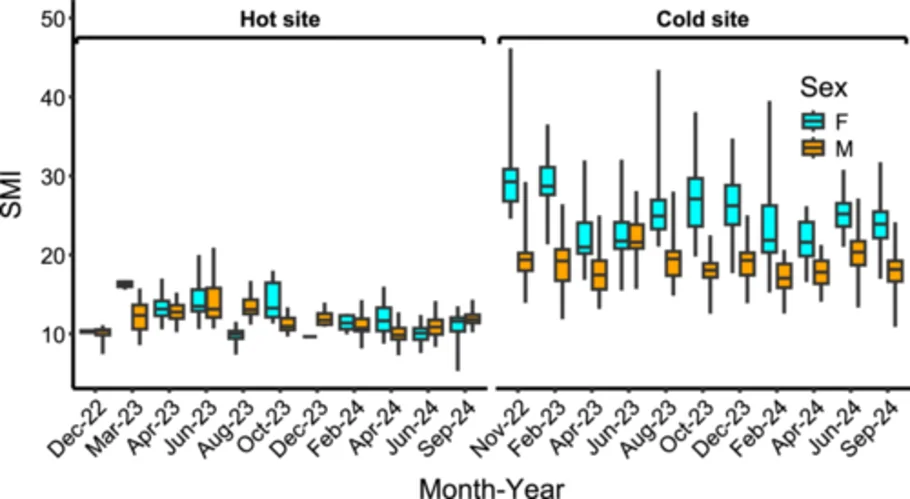
Bimonthly Scaled Mass Index (SMI) of *Rana onca* frogs compared between a hot and a cold spring, showing males (light blue) and females (orange). The boxplots indicate the 25th and 75th percentiles, with medians depicted by bold horizonal lines and extent of data by vertical lines. Sample sizes for events ranged from 9 to 56 for males and 2 to 60 for females.

## 4 Discussion

### 4.1 Annual Thermal Profiles at Hot and Cold Sites

Adult *R. onca* frogs from both the hot and cold sites, and across summer and winter samples, had similar temperature preferences; however, frogs in the wild from the two sites experienced different annual thermal profiles. Frogs at the hot site had significantly higher average T_b_ throughout the year than frogs at the cold site. This was particularly evident during summer when the differential in T_b_ averaged 5.69°C. In winter, the average difference of 2.48°C was still biologically relevant. Most physiological rates (including metabolic rate) typically increase by approximately two‐ to threefold for every 10°C rise in temperature (the temperature coefficient, Q_10_; Schmidt‐Nielsen 1990; Withers 1992). Our estimates of energy demands (derived from V̇O_2_ data) for frogs at the hot site in summer was 1.27‐fold higher than at the cold site, and 1.14‐fold higher in winter. The higher energy demands would require frogs at the hot site to consume substantially more food or, if prey is insufficient, reduce energy expenditure and rely on energy stores to support physiological maintenance at the expense of growth and reproduction (Tattersal and Ultsch 2008; Sinclair et al. 2013).

The difference in T_b_ between the hot and cold sites was not as pronounced as might have been possible given the disparity in ambient temperatures at these sites (Supporting Information S1: Table S1). In summer, the mean measured air temperature at the hot site was 11.4°C warmer than at the cold site. This difference was likely influenced by the hot site's lower elevation, hot stream, and narrow canyon topography. Frogs at the hot site showed little evidence of direct interaction with the hot stream that flows through the canyon, and only on a few occasions did we observe very short spikes (typically ≤ 35 min) in T_b_ that we interpret as brief activity in or near hot water. None of these spikes in T_b_ reached the stream temperature at those times. Rarely during the temperature preference trials did we observe frogs at temperatures approaching those of the hot stream in summer (41.82°C ± 3.43°C) or winter (33.74°C ± 3.77°C). In the trials, frogs avoided temperatures ≥ 28°C, which was roughly consistent with the mean T_b_ of frogs at the hot site in summer (27.41°C ± 1.89°C). The stream temperatures likely exceeded the physiological tolerance of *R. onca* most of the time, especially during summer. In a related study of *Anaxyrus punctatus* in the Mojave Desert, this toad avoided extreme air and surface temperatures in summer, maintaining relatively moderate T_b_ (Rausch et al. 2008). Similarly, observations of the Japanese stream tree frog (*Buergeria japonica*) in a hot spring system showed that adult frogs used relatively cold water and appeared to avoid available hot water (Komaki et al. 2020).

### 4.2 Summer Activity Differences at Hot and Cold Sites

During summer, frogs at the hot site faced high ambient air temperatures that averaged 42.41°C ± 5.65°C across both day and night. To mitigate these temperatures, frogs were likely seeking the lowest temperatures available to them, although they may have preferred even lower temperatures. We deduced this preference from observations at the cold site where frogs maintain temperatures several degrees colder than at the hot site, despite access to warmer temperatures. Currently there are few pools at the hot site cold enough for frogs to moderate body temperature during summer. Some of these pools were already reaching temperatures in late spring generally avoided by adult *R. onca*. Frogs at the hot site could potentially resist the high air temperatures over short periods through evaporative cooling (Mellanby 1941; Lillywhite 1970; Bradford 1984), but prolonged exposure without water would likely lead to rapid desiccation (Tracy et al. 2010). In response, frogs were likely constrained to cold pools, seeps, and shelters (Supporting Information S1: Figure S1), and their T_b_ reflected the temperatures of those microhabitats. This constraint also likely contributed to the lack of easily discernable differences in T_b_ at the hot site between night and day.

Most of the frogs at the cold site in summer had temperatures averaging 20.66°C ± 1.48°C for part of the day and all of the night, roughly tracking the temperature of the cold stream running through the site. At sunrise, however, their T_b_ rose with air temperature until reaching a mean of 28.28°C ± 3.81°C in the afternoon, before abruptly reverting back to temperatures reflective of the cold‐water pools. We speculate that in summer frogs at the cold site mostly stayed within the stream or in wet substrates matching stream temperatures. These frogs emerged at sunrise to bask or forage on land, and then retreated back to the stream in the afternoon as temperature increased beyond optimal (Supporting Information S1: Figure S1).

### 4.3 Differences in Winter Strategies

In cold‐temperate regions, overwintering is often perceived as a period when most ectotherms remain relatively inactive at cold temperatures, thus maintaining low physiological rates (Feder and Burggren 1992; Hillman et al. 2009; Kreitals et al. 2014). Such behavior is often interpreted as a means to conserve energy when prey is scarce and animals are reliant on energy reserves (Bradford 1983; Tattersal and Ultsch 2008). During winter, frogs at both the hot and cold sites manifested patterns in T_b_ of either relatively high or low variability. L‐V frogs at both sites appeared to thermally conform to cold waters or substrates with similar temperatures through the majority of winter. These frogs maintained mean temperatures of 11.62°C ± 1.65°C at the hot site and 11.09°C ± 1.68°C at the cold site, tracking the coldest water at each site. We suspect these L‐V frogs were able to survive on energy reserves through winter by remaining at low temperatures and maintaining limited activity, similar to many temperate anurans (Bradford 1983; Tattersal and Ultsch 2008; Hillman et al. 2009).

In contrast, H‐V frogs in winter generally had different nighttime and daytime temperatures that ranged outside of the daily variability observed in ambient air and cold‐water temperatures. At the hot site, most H‐V frogs appeared to thermally conform throughout the day to temperatures matching those of the cold pools. After sunset, however, these frogs apparently moved away from colder substrates to warmer locations probably influenced by the hot stream. At these times, T_b_ generally increased several degrees to a mean of 18.32°C ± 3.77°C, exceeding that of ambient air by several degrees. We interpret this pattern as consistent with nocturnal foraging and digestion.

At the cold site in winter, H‐V frogs mostly had nighttime T_b_ that conformed to those of the cold‐water stream, but their T_b_ increased after sunrise reaching an average of 15.19°C ± 2.30°C in the afternoon before dropping back to cold‐water temperatures. We infer that these frogs were out of the stream or other cold substrates during the day foraging and basking. This general behavioral pattern appears to have been maintained by all frogs at this site across all seasons, with the exception of L‐V frogs in winter. The apparent active warming of T_b_ at both the hot and cold sites likely increased the efficiency of foraging and digestion (Sandusky and Deban 2012; Fontaine et al. 2018), just at different times of the day (nighttime vs daytime).

What triggers overwintering behaviors (Ludwig et al. 2013; Gao et al. 2015) or factors into the selection of overwinter strategy (Tinsley 1995; Sinclair et al. 2013; Wetsch et al. 2022) remains unclear in *R. onca*. Importantly, the inversions between L‐V and H‐V modes were not periodical, indicating an absence of endogenous mechanisms typically observed in mammalian hibernators (van Breukelen and Martin 2015). Regardless of the reasons for activity during winter, the higher T_b_ of H‐V frogs implies an extra demand on energy reserves or energy acquisition over that of frogs using the more inactive strategy. The mean increase in energy demands between L‐V and H‐V modes were 1.69‐fold at the hot site and 1.66‐fold at the cold site (based on our species specific Q_10_ values).

### 4.4 Unfavorable Thermal Conditions at the Hot Spring Site

We found that the overall thermal conditions at the hot site were not favorable for *R. onca*. Frogs at the hot site appeared to have consistently experienced higher energy demands than frogs at the cold site. This would not inherently be an issue if frogs could acquire enough food to meet physiological maintenance (Gardner et al. 2011; Bazin et al. 2023). We did not assess differences in prey availability between the sites, but our measurements of body size (SVL) and condition (SMI) showed that across seasons frogs at the hot site were smaller in size, with significantly lower body condition than those at the cold site. Overall, male SMI at the hot site was 44.5% lower than at the cold site, while female SMI was 72.4% lower at the hot site.

The physiological processes associated with growth and development are temperature sensitive in ectotherms (Gardner et al. 2011); however, as temperatures increase, growth and developmental rates do not increase in parallel (Van der Have and de Jong 1996). In general, high temperatures result in rapid development towards maturation, but at a smaller size (Alves‐Ferreira et al. 2024). This process might explain the smaller size of frogs at the hot site compared to those at the cold site. Conceptually, our measure of body condition (SMI) takes into account allometric size differences (Peig and Green 2009; Ruthsatz et al. 2019), implying that frogs at the hot site had lower body condition independent of their smaller size.

Rapid development and small size associated with higher temperature have been linked to reduced adult survivorship in ectotherms (Bestion et al. 2015; Bazin et al. 2023). Possibly, the population of adult frogs at the hot site reflects lower survivorship and a resulting younger demographic than at the cold site. Male and female frogs at the hot site do not show a difference in SMI, unlike the cold site where females consistently demonstrate significantly higher SMIs across seasons. Interestingly, Saumure et al. (2022) documented several of the largest *R. onca* ever recorded at an experimental cold site. Differentiation in size and mass (subsequently SMI) between the sexes in *R. onca* likely becomes pronounced only as individuals grow with age. Conditions at the hot site may prevent frogs from reaching a threshold in size when divergence in SMI between the sexes becomes easily detected. Younger and smaller frogs at the hot site, particularly females, would have reduced capacity for reproduction (Trauth 1989; Monroe et al. 2015), which could impede population persistence and growth.

## 5 Conclusions

Our findings indicate a clear divergence in the thermal conditions for *R. onca* between the unfavorable hot site and the favorable cold site. At the hot site, most of the main thermal stream did not appear to constitute suitable habitat for the species, with water temperatures likely exceeding physiological tolerance. The stream temperatures were well outside the upper range observed for *R. onca* eggs and tadpoles (Rivera et al. 2023), and consistently exceeded the upper preferred temperatures of frogs. Adult frogs, however, appeared to occasionally use the hot stream or more likely the stream edge. Occasional brief spikes of high T_b_ noted in frog thermal profiles were interpreted as indicative of some type of interaction with the hot stream, often at times when the hot stream was the only source for the temperature increase. More moderate increases in nighttime T_b_ of some frogs in winter likely reflected nightly movements towards areas influenced by the hot stream. Also, the lowest stretch of stream loses temperature, particularly during winter, and adult frogs have been occasionally seen in those warm waters. For the most part, however, the hot stream was at best peripheral habitat for *R. onca*. This means that the amount of high‐quality habitat in the canyon was much less than the overall stream area would otherwise indicate.

As has been suspected, the few existing cold pools and seeps at the hot site appear to be critical microhabitat for adults, as well as for breeding. Without the cold substrates, the persistence of the population seems unlikely. Most of the cold pools are susceptible to sedimentation and overgrowth by emergent vegetation, and the canyon has also been prone to periodic floods that affected pool conditions. Semi‐regular maintenance of the pools appears necessary to mitigate decline or eventual loss of this critical habitat. The population at this site appears small, and increasing its abundance, thus improving resiliency, would seemingly require the creation of additional cold‐water microhabitat.

## Supporting information


Supporting File

## Data Availability

The data that support the findings of this study are openly available in figshare at https://figshare.com, reference number DOI: 10.6084/m9.figshare.29972014.
