# Supplementary material for: Higher Energy Demands and Lower Body Condition in a Hot Spring: Comparing Relict Leopard Frogs at Two Thermally Contrasting Sites
Source: J Exp Zool A Ecol Integr Physiol. 2026 Sep 1;345(10):1003–13. doi: 10.1002/jez.70129 (PMC13725228; doi:10.1002/jez.70129)
Supplement: Supplementary file 1 [file JEZ-345-1003-s001.docx]

**Table S1**. Body temperatures of adult *Rana onca* and environmental reference temperatures at a hot and a cold spring during seasons (mean ± standard deviation). Cold-water pools at the cold site reference instream pools. These pools were combined to calculate the mean stream temperature.

| Site | Source | Summer  Temperature  (°C) | Fall  Temperature  (°C) | Winter  Temperature  (°C) | Spring  Temperature  (°C) |
| --- | --- | --- | --- | --- | --- |
| Hot | Frog body | 27.40 ± 1.84 | 21.22 ± 4.35 | 15.27 ± 3.52 | 19.31 ± 4.47 |
|  | Air (shaded) | 42.41 ± 5.65 | 25.74 ± 6.57 | 12.80 ± 2.92 | 22.88 ± 6.90 |
|  | Cold pool* | 25.05 ± 1.61 | 21.06 ± 3.31 | 12.81 ± 1.77 | 16.87 ± 3.18 |
|  | Cold pool* | 30.43 ± 1.94 | 25.22 ± 3.73 | 16.39 ± 2.42 | 21.60 ± 3.71 |
|  | Warm pool | 36.27 ± 1.06 | 33.60 ± 2.37 | 28.25 ± 1.28 | 31.26 ± 2.31 |
|  | Hot stream | 41.82 ± 3.43 | 37.79 ± 3.92 | 33.74 ± 3.77 | 36.55 ± 4.52 |
| Cold | Frog body | 21.71 ± 1.49 | 17.97 ± 3.44 | 12.79 ± 2.93 | 15.45 ± 3.70 |
|  | Air (shaded) | 30.97 ± 3.12 | 17.57 ± 6.16 | 11.05 ± 3.36 | 14.38 ± 6.20 |
|  | Cold stream (mean) | 21.50 ± 1.45 | 17.86 ± 3.51 | 12.24 ± 2.05 | 15.46 ± 3.13 |
|  | Cold-water pool** | 21.78 ± 0.67 | 18.97 ± 2.79 | 13.90 ± 1.37 | 16.37 ± 2.93 |
|  | Cold-water pool*** | 21.29 ± 2.03 | 16.97 ± 3.64 | 11.82 ± 1.65 | 14.93 ± 2.75 |
|  | Cold-water pool | 20.70 ± 2.41 | 15.93 ± 3.80 | 10.90 ± 1.82 | 13.92 ± 2.84 |
|  | Cold-water pool** | 22.01 ± 2.22 | 16.74 ± 3.83 | 10.53 ± 1.74 | 14.42 ± 2.93 |

*Data from pools at the hot site shown in Figures 2 and 3.

**Data from pools at the cold site shown in Figure 3.

***Data from pool at the cold site shown in Figure 2.

**Table S2.** Estimated daily mass-specific oxygen consumption rates (V̇O_2_; ml•day^-1^•g^-1^) of adult *Rana onca* in the wild by site and season. Sample size (*n*) represent the number of daily averages, with the number of frogs contributing data shown in parentheses. V̇O_2_ measurements were interpolated to body temperature data of frogs monitored at a pair of hot and cold springs. Means are shown with ± standard deviation.

| Site | V̇O_2_ and *n* | Summer | Fall | Winter | Spring |
| --- | --- | --- | --- | --- | --- |
| Hot | Mean V̇O_2_ | 4.57 ± 0.31 | 3.75 ± 0.82 | 2.92 ± 0.55 | 3.40 ± 0.81 |
|  | Min–max V̇O_2_ | 3.39 – 5.12 | 2.26 – 5.27 | 194 – 4.43 | 1.84 – 5.06 |
|  | *n* | 368 (4) | 240 (7) | 480 (6) | 504 (11) |
| Cold | Mean V̇O_2_ | 3.60 ± 0.26 | 3.15 ± 0.59 | 2.56 ± 0.41 | 2.72 ± 0.61 |
|  | Min–max V̇O_2_ | 2.74 – 4.34 | 1.67 – 4.52 | 1.83 – 3.79 | 1.23 – 3.85 |
|  | *n* | 995 (14) | 1168 (17) | 838 (10) | 526 (10) |

**Table S3.** Annual and seasonal mean body condition (± standard deviation) of adult *Rana onca* at a hot and a cold spring estimated using Scaled Mass Index (SMI). SMI was calculated for individual frogs using body mass and snout-to-vent length. Seasonal sample sizes at the hot site ranged from 25–75 males and 11–85 females, and at the cold site ranged from 26–156 males and 33–162 females.

| Period | Hot Site Males | Cold Site Males | Hot Site Females | Cold Site Females |
| --- | --- | --- | --- | --- |
| Yearly | 11.92 ± 1.98 | 18.75 ± 2.90 | 11.81 ± 2.44 | 25.22 ± 4.45 |
| Summer | 12.99 ± 2.27 | 19.51 ± 3.18 | 11.27 ± 2.34 | 24.49 ± 3.44 |
| Fall | 11.25 ± 1.00 | 18.25 ± 2.00 | 14.30 ± 2.57 | 27.04 ± 4.30 |
| Winter | 11.48 ± 1.65 | 18.77 ± 2.86 | 11.86 ± 2.39 | 27.22 ± 5.02 |
| Spring | 11.41 ± 1.80 | 17.78 ± 2.47 | 12.41 ± 2.07 | 21.97 ± 3.22 |

**Figure S1**. Stylized depictions of activity patterns in adult *Rana onca*, as interpreted from body temperature (T_b_) data at the hot and cold sites during summer and winter. Color shading of frogs reflects the range of T_b_ going from the warmest temperatures (red frogs), to moderate temperatures (grey frogs), to cold temperatures (light blue frogs). Black double ended arrows indicate potential movement within daily periods. Movements at the hot site in winter occurred at night. In the summer, frogs at the hot site appeared to track water temperatures in the two cold-water pools, indicating that these animals stayed in or near these pools, or at seeps with similar cold temperature. At the cold site, movements occurred during the day across both seasons. In winter, frogs at both sites are depicted as having different overwintering strategies, reflecting frogs that were active (moving in and out of pools) or relatively inactive (staying within pools).
